# Possible Role of the Ca^2+^/Mn^2+^ P-Type ATPase Pmr1p on Artemisinin Toxicity through an Induction of Intracellular Oxidative Stress

**DOI:** 10.3390/molecules24071233

**Published:** 2019-03-29

**Authors:** Onnicha Pongwattanakewin, The Phyu, Suchanya Suesattayapirom, Laran T. Jensen, Amornrat N. Jensen

**Affiliations:** 1 Department of Pathobiology, Faculty of Science, Mahidol University, Bangkok 10400, Thailand; bellbaykewin@gmail.com (O.P.); 96.thephyu@gmail.com (T.P.); snookersnk14@gmail.com (S.S.); 2 Department of Biochemistry, Faculty of Science, Mahidol University, Bangkok 10400, Thailand; laran.jen@mahidol.ac.th

**Keywords:** artemisinin, oxidative stress, yeast, artemisinin resistance, Pmr1p, PfATP6

## Abstract

Artemisinins are widely used to treat *Plasmodium* infections due to their high clinical efficacy; however, the antimalarial mechanism of artemisinin remains unresolved. Mutations in *P. falciparum* ATPase6 (PfATP6), a sarcoplasmic endoplasmic reticulum Ca^2+^-transporting ATPase, are associated with increased tolerance to artemisinin. We utilized *Saccharomyces cerevisiae* as a model to examine the involvement of Pmr1p, a functional homolog of PfATP6, on the toxicity of artemisinin. Our analysis demonstrated that cells lacking Pmr1p are less susceptible to growth inhibition from artemisinin and its derivatives. No association between sensitivity to artemisinin and altered trafficking of the drug efflux pump Pdr5p, calcium homeostasis, or protein glycosylation was found in *pmr1*∆ yeast. Basal ROS levels are elevated in *pmr1*∆ yeast and artemisinin exposure does not enhance ROS accumulation. This is in contrast to WT cells that exhibit a significant increase in ROS production following treatment with artemisinin. Yeast deleted for *PMR1* are known to accumulate excess manganese ions that can function as ROS-scavenging molecules, but no correlation between manganese content and artemisinin resistance was observed. We propose that loss of function mutations in Pmr1p in yeast cells and PfATP6 in *P. falciparum* are protective against artemisinin toxicity due to reduced intracellular oxidative damage.

## 1. Introduction

**Figure 1:**
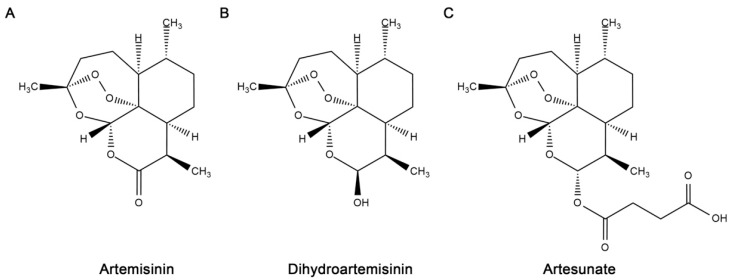
Structures of artemisinin and major derivatives prepared with ChemDraw Ultra, version 12.

Artemisinin, a natural product isolated from the leaves of the traditional Chinese medicinal plant *Artemisia annua*, has been proven to be a potent antimalarial compound [1]. Since its discovery, several derivatives of artemisinin, collectively referred to as artemisinins, have been produced including the water-soluble artesunate, the lipid-soluble artemether, and dihydroartemisinin [2]. Key structural features of artemisinins are a sesquiterpene lactone backbone and an endoperoxide bridge (Figure 1), the latter being essential for antimalarial activity [3]. Artemisinins have been widely used to treat both *Plasmodium falciparum* and *Plasmodium vivax* infections due to their high clinical efficacy (immediate onset and rapid parasite clearance) and minimal side effects [4,5,6,7]. Moreover, artemisinins remain highly effective against malaria parasites that show resistance to other classes of antimalarial medicine [8]. Combinations of artemisinin and other antimalarial drugs are utilized clinically to enhance effectiveness and minimize the development of artemisinin resistance in *Plasmodium* species [9,10,11]. Despite this strategy, artemisinin-resistant *Plasmodium* isolates have already emerged [12,13,14,15,16].

The mechanism of artemisinin action remains unresolved; however, several potential antimalarial effects have been proposed. One possible mechanism of artemisinin toxicity is by the generation of damaging reactive oxygen species (ROS) through iron-mediated endoperoxide cleavage [17,18]. However, the mechanism of artemisinin bioactivation and how ROS derived from artemisinins cause cellular damage is still a matter of debate [3,8]. Another potential mode of artemisinin action is through inhibition of *P. falciparum* ATPase6 (PfATP6), a sarcoplasmic endoplasmic reticulum Ca^2+^-transporting ATPase (SERCA). This mechanism was first proposed based on the structural similarity between artemisinins and thapsigargin, a known SERCA inhibitor [19]. PfATP6 is the only SERCA found in *P. falciparum* and limiting its activity could potentially cause growth inhibition by altering calcium homeostasis. When expressed in *Xenopus laevis* oocytes, PfATP6 activity can be inhibited by artemisinin [19]. In addition, artemisinins inhibit Ca^2+^-dependent ATPase activity in membrane fractions from *Trypanosoma cruzi* [20]. However, unlike the case for thapsigargin, artemisinin does not inhibit purified PfATP6 [21]. This finding suggests that artemisinins may not directly inhibit PfATP6 in vivo but instead exert their effect on PfATP6 through an indirect mechanism. Interestingly, a mutation in PfATP6 in parasite field isolates from French Guiana and Senegal was reported to associate with reduced susceptibility to artemisinin derivatives [13,22]. However, how PfATP6 inhibition participates in the antimalarial action of artemisinin remains unclear.

The yeast *Saccharomyces cerevisiae* is a powerful model system for identifying genes and biochemical pathways involved in the action of drugs [23,24,25] and artemisinins are active against this model organism [26]. The *S. cerevisiae* Ca^2+^-ATPases, vacuolar localized Pmc1p and Golgi localized Pmr1p [27], appear to be targets for artemisinin [28], providing an attractive cellular model to investigate the mechanism of PfATP6 inhibition by artemisinins. In this study, we examined the role of *S. cerevisiae* Pmr1p on the toxicity of artemisinin and its derivatives. Our analysis demonstrated that cells lacking Pmr1p are less susceptible to artemisinins. The investigation into the underlying cause of artemisinin resistance in *pmr1*Δ cells found no alteration in the localization or accumulation of the multi-drug transporter Pdr5p as well as no association between defects in calcium homeostasis or protein glycosylation and sensitivity to artemisinin. These findings suggest that the artemisinin resistance observed in *pmr1*∆ yeast is not due to defects in processes known to be associated with loss of Pmr1p function. Our analysis has revealed that *pmr1*∆ cells exhibit elevated basal levels of ROS, which are not further increased following artemisinin exposure. This is in sharp contrast to WT cells, where artemisinin treatment produced a significant increase in intracellular ROS. It is possible that Pmr1p may play a role in ROS generation or accumulation from artemisinin. We propose that the loss of function in Pmr1p or PfATP6 results in reduced artemisinin sensitivity due to insufficient ROS being generated to cause significant cellular damage.

## 2. Results

### 2.1. Yeast Cells Lacking Pmr1p Display Resistance to Artemisinin and Its Derivatives

**Figure 2:**
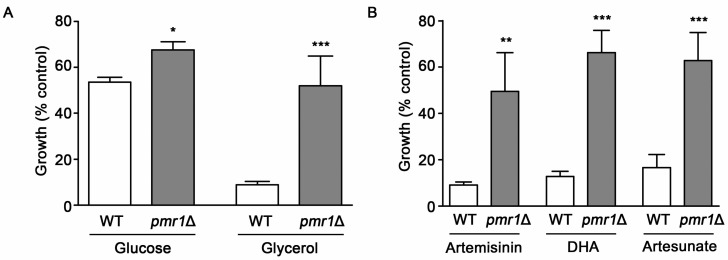
Yeast cells lacking Pmr1p are less susceptible to artemisinin and its derivatives. (**A**) WT and *pmr1*∆ cells were cultured in synthetic complete media supplemented with either 2% glucose (SC) or 2% glycerol (SCG) as a carbon source with vehicle or 10 μM artemisinin and growth was monitored at 24 h after treatment. (**B**) WT and *pmr1*∆ cells were cultured in SCG medium as described in (**A**) and treated with vehicle, 10 μM artemisinin, 2 μM dihydroartimisinine (DHA), or 10 μM artesunate for 24 h. Percent growth compared to untreated controls for each strain is shown. Results are from three independent experiments; values are the mean + SD with differences between strains analyzed using two-tailed unpaired Student’s *t*-test; ***P < 0.001.

The *P. falciparum* sarcoendoplasmic reticulum Ca^2+^ ATPase6 (PfATP6) has been suggested as one of the key targets of the toxic actions of artemisinin [19]. Consistent with this proposal, mutations in PfATP6 have been shown to be associated with decreased sensitivity to artemisinin [13,29]. How PfATP6 is involved in modulating artemisinin toxicity has not been clearly elucidated. Analysis of yeast cells lacking the Ca^2+^/Mn^2+^ P-type ATPase Pmr1p, a homolog of *Plasmodium* PfATP6, has the potential to inform regarding possible mechanisms that limit artemisinin toxicity. In WT yeast, artemisinin toxicity is enhanced under growth conditions that require respiration [26]. However, it is not known if altered artemisinin sensitivity from PfATP6 mutations is linked to respiratory activity. Our analysis of artemisinin sensitivity for WT yeast in respiratory (glycerol) and fermentative (glucose) medium gave results consistent with previous reports, indicating increased artemisinin sensitivity under respiratory conditions [26]. The *pmr1*∆ strain exhibited reduced susceptibility to artemisinin, compared to WT yeast, in both respiratory and fermentative medium (Figure 2A). Due to the higher levels of artemisinin sensitivity seen in WT cells with respiratory medium, these conditions were used in subsequent experiments. Reduced toxicity to artemisinin derivatives dihydroartemisinin and artesunate were also observed in *pmr1*∆ yeast (Figure 2B), suggesting that artemisinin derivatives containing an endoperoxide bridge may require the presence of Pmr1p for their toxic actions.

### 2.2. The Slower Growth Rate of pmr1∆ Cells Does Not Appear Sufficient to Reduce Artemisinin Sensitivity

**Figure 3:**
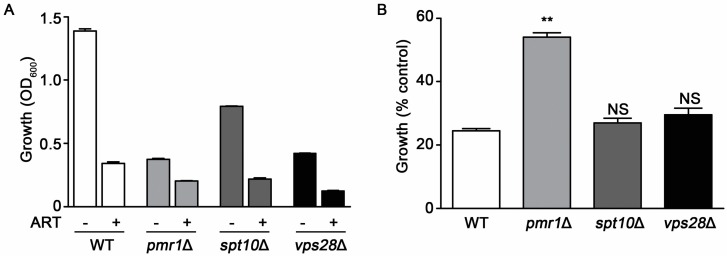
Slow growth is not directly associated with artemisinin sensitivity. Growth profiles for WT, *pmr1*∆, *spt10*∆, and *vps28*∆ cells in SCG medium monitored at 24 h after treatment with vehicle or 10 μM artemisinin. (**A**) OD600 nm values and (**B**) percent growth compared to untreated controls for each strain. Results are from two independent experiments; values are the mean + SD with differences between WT and mutant strains analyzed using two-tailed unpaired Student’s *t*-test; **P < 0.01.

The growth rate of cells can alter susceptibility to several drugs. Slowly growing cells often survive exposure to drugs or other environmental stresses better than those replicating quickly [30]. Yeasts deleted for *PMR1* exhibit substantial growth reduction compared to WT cells, which may contribute to their decreased sensitivity to artemisinin. To examine this possibility, we evaluated artemisinin sensitivity in two other deletion strains that exhibit reduced growth rates, *spt10*∆ and *vma28*∆. *SPT10*, encodes a histone H3 acetylase [31], while Vps28p is a component of the ESCRT-I complex [32]. Compared to the WT strain, *pmr1*∆ cells proliferate poorly with the *vps28*∆ and *spt10*∆ strains also showing reduced growth rates. Following treatment with artemisinin both *spt10*∆ and *vps28*∆ cells display a similar level of growth inhibition as the WT strain, indicating that these strains are sensitive to the effects of artemisinin toxicity (Figure 3A). To more easily observe the change in growth of strains when challenged with artemisinin, growth relative to vehicle-treated cells (% control growth) is also presented (Figure 3B).

### 2.3. Localization and Accumulation of the Drug Efflux Transporter pdr5p Is Not Altered in pmr1∆ Cells

**Figure 4:**
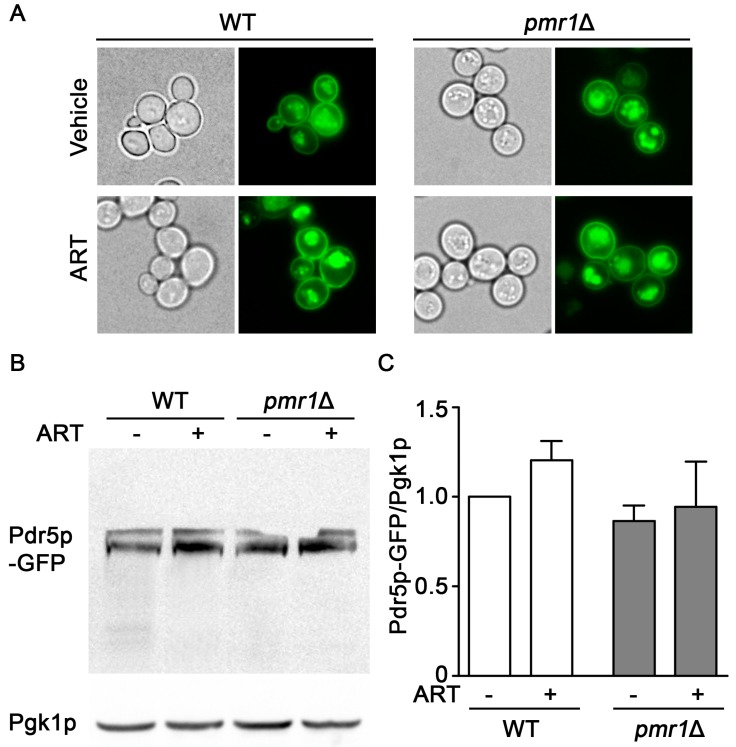
Localization and accumulation of the multi-drug transporter Pdr5p are similar in *pmr1*Δ and WT cells. WT and *pmr1*Δ yeast transformed with a plasmid expressing *PDR5*-GFP fusion driven by the constitutive *PGK1* promoter were grown overnight in SCG media with vehicle or 10 μM artemisinin. (**A**) Cellular localization of Pdr5p-GFP was observed by fluorescence microscopy at 60x magnification. (**B**) The abundance of Pdr5p-GFP was monitored with immunoblots using Pgk1p levels as the loading control. (**C**) Quantitation of Pdr5p-GFP results are normalized to the intensity of Pgk1p with levels normalized to WT = 1. Values are mean + SD (*n* = 2). No statistical difference between samples was observed using Student’s *t*-test.

Increased expression of the plasma membrane multi-drug resistant transporter Pdr5p is associated with resistance to multiple drugs and functions by facilitating drug efflux from the cell [33]. Expression of Pdr5p following exposure to artemisinin appears critical for resistance to this drug; in addition, *pdr5*∆ cells are sensitized to artemisinin [34]. Pdr5p is transported to the plasma membrane through the secretory pathway and, when targeted for degradation, is delivered to the vacuole through transport vesicles [35]. *PMR*1 deletion mutants are defective in several functions in the secretory pathway, which can result in longer residency times for plasma membrane proteins [36,37]. This effect of *PMR1* deletion on plasma membrane proteins raised the question of whether aberrant accumulation or localization of Pdr5p may contribute to increased artemisinin resistance. Examining Pdr5p localization using a GFP fusion indicated similar localization patterns in both WT and *pmr1*∆ yeast, regardless of artemisinin exposure (Figure 4A). The abundance of Pdr5p-GFP was also examined using immunoblots and no significant change in Pdr5p levels was observed between WT and *pmr1*∆ cells, regardless of artemisinin exposure (Figure 4B,C). It thus seems unlikely that artemisinin resistance in *pmr1*Δ cells is due to the increased Pdr5p-mediated cellular efflux of artemisinin.

### 2.4. Deletions of Genes Encoding Proteins Involved in Ca^2+^ homeostasis or Protein Glycosylation Does Not Promote Artemisinin Resistance

**Figure 5:**
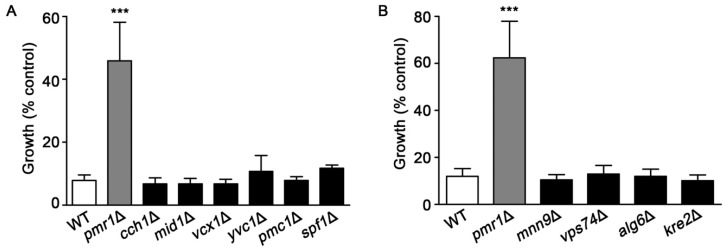
Yeast mutants lacking proteins involved in calcium homeostasis or protein glycosylation are not resistant to artemisinin. WT, *pmr1*Δ, and strains deleted for genes encoding proteins functioning in (**A**) cellular calcium homeostasis; *cch1*Δ, *mid1*Δ, *vcx1*Δ, *yvc1*Δ, *pmc1*Δ, and *cod1*Δ or (**B**) protein glycosylation; *mnn9*Δ, *vps74*Δ, *alg6*Δ, and *kre2*Δ were cultured in SCG media with vehicle or 10 μM artemisinin. Growth was assessed at 24 h by measuring OD_600_. Results are from three independent experiments; values are the mean + SD. ***P < 0.001 were determined using one-way ANOVA.

Pmr1p is a key transporter for uptake of Ca^2+^ into the Golgi, which is important for maintaining the Ca^2+^ homeostasis of yeast cells [38,39]. We investigated whether the loss of other proteins that participate in Ca^2+^ transport would alter sensitivity to artemisinin. Yeast strains examined were deleted for genes encoding the plasma membrane Cch1p/Mid1p channel complex [40,41]; vacuolar Ca^2+^ channels Vcx1p and Pmc1p, and Yvc1p [42,43,44,45]; and the ER-associated P-type ATPase Spf1p [46]. Among the yeast strains defective in Ca^2+^ homeostasis, only cells lacking Pmr1p showed resistance to artemisinin (Figure 5A).

Yeast *pmr1*∆ mutants also exhibit defects in protein glycosylation [36,37], which could be associated with enhanced resistance to artemisinin. Several yeast mutants, in addition to *pmr1*∆, have significant protein glycosylation defects, these include *mnn9*Δ, *vps74*Δ, *alg6*Δ, and *kre2*Δ [47,48,49,50,51]. As shown in Figure 5B, deletion strains with protein glycosylation defects did not exhibit the increased resistance to artemisinin seen in cells lacking Pmr1p. Together, these results suggest that altered calcium trafficking or defects in protein glycosylation are not sufficient to reduce toxicity from artemisinin.

### 2.5. Increased Intracellular Oxidation Is Not Observed in pmr1∆ Yeast Following Artemisinin Exposure

**Figure 6:**
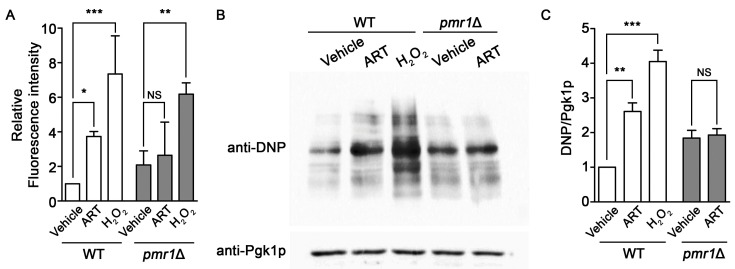
Yeast *pmr1*∆ mutants exhibit increased basal intracellular ROS and protein oxidation, which is not elevated following artemisinin exposure. WT and *pmr1*∆ cells were cultured in SCG media with vehicle or 10 μM artemisinin for 24 h. (**A**) ROS levels were monitored using DCFH-DA. The fluorescence intensity of the samples was normalized to protein content with levels set to WT = 1. Results are from two independent experiments; values are the mean + SD. Differences between strains were analyzed using the two-tailed unpaired Student’s *t*-test; ***P < 0.001. (**B**) Protein carbonyls from the oxidation of protein side chains were monitored in whole cell lysates following derivatization with DNPH (2,4-dinitrophenylhydrazine). DNP-adducts were detected by immunoblotting with an anti-DNP antibody (upper panel) and the cytosolic protein Pgk1p (lower panel) was monitored as a loading control. (**C**) Quantitation of protein carbonylation results are normalized to the intensity of Pgk1p with levels normalized to WT = 1. Values are mean + SD (*n* = 2). * *P >* 0.05, ** *P >* 0.01, ***P < 0.001, determined using Student’s *t*-test.

Artemisinin can promote the production of intracellular ROS and oxidative damage, resulting in cellular injury following drug exposure [26,52]. *PMR1* deletions are capable of protecting against oxidative damage in yeast lacking Cu/Zn superoxide dismutase [53], suggesting the possibility of a similar effect of this mutant on artemisinin generated ROS. Intracellular ROS levels, determined using DCFH-DA, clearly show increased ROS accumulation in WT cells exposed to artemisinin or hydrogen peroxide. ROS production in *pmr1*∆ yeast was not elevated following artemisinin treatment; although, ROS levels in the untreated *pmr1*∆ strain was elevated compared to the WT control. Treatment with hydrogen peroxide was capable of increasing ROS levels in the *pmr1*∆ mutant to levels greater than that seen in untreated cells or following artemisinin exposure (Figure 6A). This indicates that the lack of ROS production in pmr1∆ yeast exposed to artemisinin is not due to saturation of the detection system. In addition, the ability of hydrogen peroxide to increase ROS levels suggests that deletion of *PMR1* is not protective against ROS generation from all sources. Analysis of oxidative damage to proteins, through monitoring of protein carbonylation, revealed similar results as obtained with ROS determination. Protein carbonylation in WT cells was substantially increased following exposure to artemisinin or hydrogen peroxide. Consistent with ROS levels, similar quantities of carbonylated proteins were detected in untreated *pmr1*∆ yeast and cells exposed to artemisinin; protein carbonyl levels were elevated relative to untreated WT cells (Figure 6B,C). It appears that the production of damaging intracellular ROS following artemisinin exposure may require the activity of Pmr1p.

### 2.6. Elevated Intracellular Mn Is Not Protective against Artemisinin Toxicity

**Figure 7:**
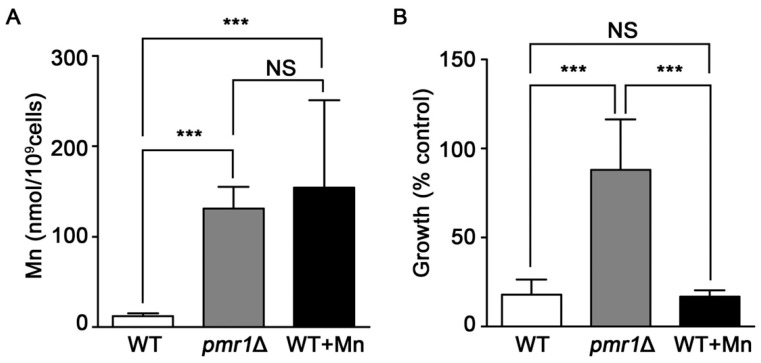
Elevated levels of intracellular Mn do not appear to result in reduced artemisinin susceptibility. (**A**) WT, *pmr1*Δ, and WT treated with 12 mM Mn were grown in SCG media for 24 h and the intracellular Mn levels were measured with atomic absorption spectroscopy. Results are from duplicate measurements (**B**) WT, *pmr1*Δ, and WT pre-treated with 12 mM Mn for 24 h were cultured in SCG media with vehicle or 10 μM artemisinin and growth was measured by OD_600_ at 24 h. Percent of growth compared to vehicle is shown. Results are from three independent experiments; values are the mean + SD. ***P < 0.001 determined using one-way ANOVA.

Yeast deleted for *PMR1* exhibit high levels of intracellular Mn ions [53], which can provide protection against oxidative stress by functioning as ROS scavengers when complexed with phosphate or other small molecules [53,54,55,56]. It is possible that the elevated accumulation of Mn in *pmr1*Δ cells is protective against oxidative injuries caused by artemisinin, limiting the toxicity of the drug. We addressed this possibility by growing WT yeast in medium supplemented with sufficient Mn to produce similar intracellular Mn levels to that found in *pmr1*Δ cells (Figure 7A). However, WT yeast with elevated levels of intracellular Mn do not display an increase in artemisinin resistance (Figure 7B). Thus it appears that elevated intracellular Mn is not sufficient to protect against artemisinin toxicity, indicating that another mechanism is responsible for limiting artemisinin sensitivity in *pmr1*∆ cells.

## 3. Discussion

Artemisinins have been used for the treatment of malaria for many decades; however, the precise mechanism for its antimalarial action is still in question. One of the proposed targets of artemisinin is PfATP6, the sarcoendoplasmic reticulum Ca^2+^ ATPase from *Plasmodium falciparum* [19]. However, the limited genetic tools available for *Plasmodium* species makes the use of model systems a valuable alternative for the investigation into the causes of artemisinin toxicity. In this study, we utilized *S. cerevisiae* to examine the contribution of Pmr1p, a functional homolog to PfATP6, in artemisinin toxicity. Consistent with a previous report on the reduced susceptibility of yeast lacking both PfATP6 homologs Pmr1p and Pmc1p to artemisinin [28], we observed that yeast lacking Pmr1p are much less sensitive to artemisinin than WT cells. Additionally, our analysis demonstrated that the *pmr1*∆ strain was also less sensitive to artemisinin derivatives, dihydroartemisinin, and artesunate, which contain the endoperoxide bridge. This suggests that loss of Pmr1p is likely protective against artemisinin toxicity exerted through the endoperoxide structure essential for the antimalarial activity of artemisinins.

To better understand the molecular processes that lead to reduced artemisinin sensitivity in *pmr1*∆ cells, several possible protective mechanisms were examined. One of the major effects from loss of Pmr1p is impaired N- and O-linked glycosylation, resulting in protein sorting and processing defects in the secretory pathway [37]. Increased sorting and stabilization of Gap1p at the plasma membrane is observed in *pmr1*∆ cells [57] and accumulation of other transporters may be similarly enhanced. Expression of Pdr5p, a major ATP-binding cassette (ABC) drug efflux pump, contributes to artemisinin resistance by facilitating the export of this drug [34]. Plasma membrane localization of Pdr5p is required to facilitate artemisinin tolerance. Yeast with impaired ER to Golgi trafficking exhibit mislocalization and decreased abundance of Pdr5p, leading to artemisinin sensitivity [58]. However, the localization of the Pdr5p transporter was not altered either by deletion of *PMR1* or exposure to artemisinin. Enhanced plasma membrane localization of this drug efflux transporter does not appear to be a major factor in artemisinin resistance in *pmr1*∆ yeast. Consistent with this finding, deletion strains with disturbances in glycosylation or protein sorting (*mnn9*Δ, *vps74*Δ, *alg6*Δ, and *kre2*Δ) do not show enhanced artemisinin resistance compared to WT yeast. Over-expression of *PDR5* can also promote artemisinin resistance [34,58]; however, the accumulation of Pdr5p-GFP was not significantly different between WT and *pmr1*∆ cells treated with either vehicle or artemisinin. Together, these results indicate that defects in Pdr5p sorting, processing, or accumulation are not the major underlying cause promoting artemisinin resistance in cells deficient for Pmr1p.

Calcium is involved in many cellular processes and sustained increases in intracellular calcium have been associated with drug resistance [59]. In the absence of Pmr1p, cytosolic Ca^2+^ concentrations are elevated due to decreased transport of this cation into the Golgi [60]. The contribution of Pmr1p to calcium homeostasis could be a factor in the decreased artemisinin sensitivity seen in *pmr1*∆ yeast. However, artemisinin toxicity in yeast with deletion of genes encoding calcium transporters localized to the plasma membrane (*cch1*Δ and *mid1*Δ) [40,41], vacuole (*vcx1*Δ, *yvc1*Δ, and *pmc1*Δ) [43,45,61], or ER (*spf1*Δ) [46] did not produce enhanced resistance to artemisinin. It appears that more than altered calcium homeostasis is required to facilitate resistance to artemisinin.

Production of ROS following artemisinin exposure has been well documented and cleavage of the endoperoxide bridge appears to be a key event that facilitates cellular oxidative damage [62,63,64]. Evaluation of ROS production and oxidative damage to proteins revealed a substantial increase in both of these parameters in WT yeast following artemisinin exposure. In contrast, *pmr1*∆ cells exhibit an elevation in ROS production and protein oxidative damage compared to untreated WT cells, but no apparent increase when challenged with artemisinin. Although an upward trend for ROS levels in *pmr1*∆ yeast exposed to artemisinin was seen, this difference was not statistically significant. Even though *pmr1*∆ yeast do not exhibit a statistically significant increase in ROS following artemisinin exposure, these cells are experiencing elevated basal levels of ROS. The cause of the elevated basal ROS levels in *pmr1*∆ cells is not clear but may be related to impaired calcium trafficking [65].

Yeast deleted for *PMR1* can bypass the oxygen sensitivity of cells lacking Cu/Zn superoxide dismutase (*sod1*∆), an important enzyme in the detoxification of oxygen radicals [53]. The ability of *PMR1* deletion to protect *sod1*∆ cells from oxygen toxicity is linked to the over-accumulation of intracellular manganese ions. Supplementation of WT yeast with manganese can also provide enhanced protection against oxidative insults through the formation of Mn-dependent ROS scavenging complexes [56]. However, providing WT yeast with manganese did not protect against artemisinin toxicity, suggesting that artemisinin resistance in cells lacking Pmr1p does not simply arise as a result of an elevated intracellular concentration of manganese that may act as antioxidant molecules. Examination of a *PMR1* knockdown in *Caenorhabditis elegans* has also suggested a role for Mn-based antioxidant complexes in protecting against oxidative stress. In addition, it was proposed that cytosolic Mn may alter transcriptional activity, increasing expression of antioxidant enzymes, such as mitochondrial Mn-superoxide dismutase [66]. Currently, it is not known how the transcription pattern of yeast is altered by the deletion of *PMR1*. Although, the activity of yeast Mn-Sod2p in not elevated in *pmr1*∆ cells [67]. It is possible that the protection against artemisinin toxicity provided by deletion of *PMR1* in yeast is mediated through altered expression or activity of antioxidant enzymes.

The elevated ROS accumulation in *pmr1*∆ cells may be functioning to promote an adaptive response that is protective against the effects of artemisinin exposure. Pretreatment with sub-lethal stress can in many cases lead to greater resistance to the lethal effects of subsequent treatment of higher concentrations of pro-oxidant molecules [68,69,70]. Alternatively, it is possible that a direct interaction between artemisinin and Pmr1p may promote ROS production. While the cause of elevated ROS levels in the *pmr1*∆ strain is not clear, Pmr1p activity is required to prevent higher than normal basal levels of ROS. Based on this observation, inhibition of Pmr1p by artemisinin would lead to enhanced oxidative stress similar to that seen in *pmr1*∆ cells. Deletion of *PMR1* may prevent additional ROS formation, above the already elevated basal levels, by removing a target of artemisinin action. An indirect mechanism in which Pmr1p facilitates ROS generation is also possible, although how Pmr1p could modulate ROS production from artemisinin remains unknown. Overall, our results herein suggest a possible role for Pmr1p in the generation of damaging ROS from artemisinin. If this proposed role for Pmr1p is proven to be the case, this may explain the reduced artemisinin of *P. falciparum* containing mutations or polymorphisms in PfTAP6.

## 4. Materials and Methods

### 4.1. Yeast Strains, Plasmid, and Culture Conditions

The strains used in this study are isogenic to the haploid strain BY4742 (*Mat α, leu2∆0, lys2∆0, ura3∆0, his3∆1*). Single gene deletion strains, *pmr1*Δ, *cch1*Δ, *mid1*Δ, *vcx1*Δ, *yvc1*Δ, *pmc1*Δ, *spf1*Δ, *mnn9*Δ, *vps74*Δ, *alg6*Δ, *kre2*Δ, *spt10*∆, and *vps28*∆ were purchased from Open Biosystems, Inc. (Layafette, CO, USA). Plasmids pWC018 expressing *PDR5*-GFP driven by the *PGK1* promoter has been described previously [58]. Cells were maintained at 30 °C on a synthetic complete medium supplemented with either 2% glucose (SC) or 2% glycerol (SCG). Yeast transformations were performed using the lithium acetate procedure [71]. Transformants were selected using an SC medium lacking uracil. Artemisinin and derivatives (AdooQ Biosciences, Irvine, CA, USA) were dissolved in 100% ethanol.

### 4.2. Fluorescence Microscopy

The localization of Pdr5p was examined by employing a plasmid overexpressing GFP-tagged Pdr5p (PWC018). Cells containing GFP-Pdr5p were cultured overnight with or without artemisinin treatment at 30 °C to achieve an OD_600_ value of 1 in the SCG liquid medium lacking uracil. The intracellular localization of GFP-tagged Pdr5p was visualized in live cells and imaged as previously described [72]. Brightfield images were also captured to visualize cell area. Fluorescence was viewed using an Olympus BX53 fluorescent microscope at a magnification of 60× (Olympus Bioimaging Center, Mahidol University).

### 4.3. Measurement of Intracellular ROS Levels

2,7-Dichlorofluorescein diacetate (DCFH-DA) (Sigma, St. Louis, MO, USA) was used to measure intracellular ROS levels in response to artemisinin treatment. Yeast were treated with vehicle, artemisinin, or H_2_O_2_ for 24 h, followed by incubation with 10 μM DCFH-DA for one hour. Cell lysates, prepared in PBS buffer with glass bead extraction, were utilized for the measurement of fluorescence intensity as this technique has been shown to give higher sensitivity compared to using intact yeast [73,74]. The fluorescence signal was measured with an excitation at 490 nm and emission at 535 nm using a Spark 10M multimode microplate reader. Fluorescence intensity was normalized to the protein concentration of each sample. Results are from three independent experiments.

### 4.4. Immunoblots and Protein Carbonylation Analysis

Cultures for determination of Pdr5p-GFP abundance were grown in SCG medium. Extracts were generated using NaOH lysis with TCA precipitation as previously described [35]. Immunoblots were probed with anti-GFP (Santa Cruz Biotechnology, Dallas, TX, USA) or anti-Pgk1 (Abcam, Cambridge, MA, USA) antibody at a dilution of 1:5000. Carbonylated proteins were detected following derivatization with 2,4-dinitrophenylhydrazine (DNPH) as previously described [75]. Lysates were prepared from cells grown in SCG medium treated with vehicle, 10 μM artemisinin, or 5 mM H_2_O_2_ for 24 h. Twenty-five micrograms of protein from each sample were reacted with DNPH for 15 min at 25 °C. DNP-derivatized proteins were detected using immunoblots as described previously using an anti-DNP antibody (Merck Millipore, Burlington, MA, USA) at a dilution of 1:5000 [76]. Visualization of immunoblots utilized an HRP-conjugated secondary antibody and ECL detection (Merck Ltd.) with a G:Box Chemi XL1.4 chemiluminescence imaging system (Syngene, Frederick, MD, USA). Quantitation of protein intensity utilized ImageJ 1.45S software (National Institute of Health, Bethesda, MD, USA) [77].

### 4.5. Measurement of Intracellular Manganese

WT yeast, WT supplemented with 12 mM manganese (Mn), and *pmr1*Δ cells were cultured in SCG media for 24 h. Cells were then collected and washed with 1ml Tris HCl with EDTA pH 6.5 and Type 1 water. Mn levels were measured using graphite furnace atomic absorption spectroscopy (PinAAcle 900T; PerkinElmer, Waltham, MA, USA) and reported as nmol Mn/10^9^ cells.

### 4.6. Statistical Analysis

Experimental data are reported as the mean + the standard deviation (SD). Significant differences between or among groups are indicated with, **P < 0.01 and ***P < 0.001. Data were analyzed by one-way ANOVA with post hoc Tukey test or Student’s *t*-test as appropriate.

## 5. Conclusions

Overall, our analysis has revealed an interesting role for *S. cerevisiae* Pmr1p, a functional homolog of PfATP6, in modulating toxicity from artemisinins. Deletion of *PMR1* appears to prevent the induction of oxidative stress from artemisinin exposure through a mechanism unrelated to enhanced accumulation of manganese or altered calcium homeostasis. Further analysis into Pmr1p/PfATP6-mediated artemisinin toxicity may allow for better understanding into the development of drug resistance in *Plasmodium* species.
